# Positive symptoms and their associations with life and trauma events among young adults in a first-episode psychosis clinic: qualitative analysis

**DOI:** 10.1192/bjo.2025.10802

**Published:** 2025-08-15

**Authors:** Alix-Anne Ternamian, Mathilde Marchal, Julie Haesebaert, Frédéric Haesebaert

**Affiliations:** Centre Hospitalier le Vinatier, Bron, France; Hospices Civils de Lyon, Pôle Santé Publique, Service Recherche et Epidémiologie Cliniques, Lyon, France; Université Claude Bernard Lyon 1, INSERM U1290, Research on Healthcare Performance RESHAPE, Lyon, France; Université Claude Bernard Lyon 1, CNRS, INSERM, Centre de Recherche en Neurosciences de Lyon CRNL U1028 UMR5292, PSYR2Bron, France

**Keywords:** First episode psychosis, positive psychotic symptoms, trauma, qualitative, experience

## Abstract

**Background:**

Trauma plays a critical role in psychosis, but the nature of the relationship between specific symptoms and trauma history remains unclear.

**Aims:**

The aim of the study was to explore the experience of positive symptoms and their association with trauma and life events from the perspective of patients with first-episode psychosis (FEP).

**Method:**

Seventeen participants who were enrolled in an FEP programme participated in a qualitative interview examining their life and trauma events, the onset of their symptoms, their experience of positive symptoms and their perceived associations between symptoms and life and trauma events. The interview was based on a semi-structured interview of six main questions and follow-up questions. Participants also completed the Trauma and Life Experiences Checklist (TALE), and were asked about the relevance of the whole interview. Thematic content analysis, exploratory cluster analysis and matrix queries coding were performed.

**Results:**

Fifteen participants described the experience of psychotic symptoms as distressing or traumatic. Eleven participants attributed the onset of positive psychotic symptoms to trauma and life events. Ten participants described explicit thematic associations between their symptoms and trauma and life events. Twelve participants evaluated the interview as relevant and helpful.

**Conclusions:**

Our findings give insight into the lived experience of positive symptoms and potential psychological interventions valuing causal theories of participants and the association with life and trauma events.

A history of trauma, particularly childhood adversity, among patients is a recognised risk factor for developing psychosis.^1^

Although evidence suggests that childhood trauma is associated with brain alterations in psychosis,^2^ the association between psychotic symptoms and trauma history is not clearly understood at both clinical and pathophysiological levels.

A history of trauma has been preferentially associated with positive psychotic symptoms, notably in patients with first-episode psychosis (FEP).^3^ However, the links between specific types of positive psychotic symptoms and specific types of traumas remain unclear. Some studies found associations between them, in particular hallucinations and/or delusions.^4^ Bentall and colleagues found associations between a history of childhood rape and auditory-verbal hallucinations, between institutionalisation and paranoid ideation, and between childhood physical abuse and auditory-verbal hallucinations and paranoid ideation.^5^ In other studies, no relationship between particular types of trauma and psychotic symptoms was found.^6^ Some authors reported on the impact of multiple trauma exposure on psychosis rather than single events.^7^

Few qualitative studies were conducted in the field. They found thematic associations between trauma history and positive symptoms,^8^ trauma and auditory hallucinations,^9^ and trauma and hallucinations.^10^

Few qualitative studies have investigated patients’ views on the association between positive psychotic symptoms and traumatic history.^11–13^ To our knowledge, none were specifically conducted in patients with FEP.

The current study aimed to describe the experience of positive psychotic symptoms and its association with different types of life and trauma events, considering the timing of exposure from the perspective of patients who have experienced FEP.

## Method

Semi-structured interviews were conducted to gather the patients’ perspective on positive symptoms and links to their life and trauma history. Qualitative studies are particularly relevant when a topic is still relatively unexplored. They enable us to generate hypotheses that can then further be tested quantitatively. They provide access to detailed individual experience in order to understand a phenomenon.

### Setting and sample

Participants were recruited via the ‘PEP’S’ FEP programme in Greater Lyon (France). Inclusion criteria were as follows: to have experienced FEP with a diagnosis made by an FEP-specialised psychiatrist, including those induced by post-traumatic stress disorder and those with or without affective symptoms; to speak and read French; to be aged between 18 and 35 years and to consent to the study. Exclusion criteria were: having an intellectual disability (<70 IQ on premorbid intelligence estimate), patient refusal, adults under guardianship or tutorship, disorganisation greater than 4 on the Comprehensive Assessment of At-Risk Mental States (CAARMS),^14^ an ongoing suicidal crisis, and hearing or vision problems that could compromise the interview process. All participants meeting the inclusion criteria and involved in the PEP’S programme were considered for inclusion. All eligible participants were contacted by their case manager during their appointments or shortly before discharge from hospital. Recruitment took place on an ongoing basis until the appropriate sample was reached. Analysis was performed continuously, and recruitment was stopped when data saturation was reached.

### Ethical considerations

The authors assert that all procedures contributing to this work comply with the ethical standards of the relevant national and institutional committees on human experimentation and with the Helsinki Declaration of 1975, as revised in 2013. All procedures involving human patients were approved by an Institutional review Board (Comité de Protection des Personnes Ile de France VII, approval number 2022-A01780-43). All participants were informed of the qualitative aspects of the interview, the recording and the themes that would be covered. They gave their consent before their inclusion in the study. Verbal consent was witnessed and formally recorded.

### Population data collection

For each participant, the following sociodemographic characteristics and psychosis history were collected: gender, age, education, minority ethnic group, being a first- or second-generation migrant, comorbidities and associated symptoms, diagnosis, duration of involvement in FEP programme, family history, medical and psychiatric history, and history of substance misuse. Participants were also asked about the presence of a specific context of onset of their episode or precipitating factors. Participants were interviewed for 60 to 180 min by A.-A.T., a female psychiatric resident trained in CAARMS interviewing and qualitative analysis. Before the beginning of the interview, a communication contract was presented, and the participant was asked for their consent for the study. During the interview, the interviewer first verified and completed sociodemographic information. Next, the participant and interviewer completed the Trauma and Life Experiences Checklist (TALE). The TALE is a 22-item scale that can be either self- or researcher-administered. It is designed for routine trauma screening in psychosis services. It includes a list of common traumatic or stressful life events, as well as an item where participants can discuss traumas not covered in the questionnaire. It has the advantage of including psychosis-specific potentially traumatic events (the experience of psychotic symptoms, hospital admission and unusual behaviours). It has moderate psychometric acceptability overall, with excellent convergent validity and reliability for sexual abuse.^15^

The researcher-administered TALE questionnaire was not audio recorded, as this was often the first meeting with the participant and the questions were very personal. The results of the TALE were collected in a Microsoft Excel document and detailed notes were made with the participants’ answers to each question. Third, the interviewer conducted an individual qualitative interview that was audio recorded with the participant’s consent. The qualitative semi-structured interview consisted of six main questions with follow-up (see Appendix 1: semi-structured interview guide). The interview was stopped when all questions were answered, including the follow-up questions. Interview duration then varied depending on the verbatim lengths for each patient or the global speech speed. This allowed each participant to specify the context of onset, describe the positive psychotic symptoms experience, detail the characteristics of the positive psychotic symptoms and develop their opinion about the origin of the symptoms and an eventual link between trauma and life events (see Table 1). The questions exploring positive psychotic symptoms and the experience of positive psychotic symptoms were derived from the CAARMS. The CAARMS is a semi structured interview with a French validation that assesses and details positive psychotic symptoms, in particular attenuated positive psychotic symptoms.^14^

**Table 1:** Theme 4: Thematic associations between specific positive psychotic symptoms and specific life and trauma events, with quotes from the perspective of participants

Participant	Trauma and life events in the association	Positive psychotic symptoms in the association	Quotes of the thematic association
1	Sexual abuse before 16 years old by her father Physical abuse Death threat by her father Emotional abuse	Olfactory hallucinations Tactile hallucinations Persecutory delusions Ideas of life threat Esoteric delusions Grandiose ideas	*‘Frankly, the smell of rape is nauseating, extremely strong, somewhere between burnt rice and rotten eggs.’* *‘When you’ve been the victim of a crime, you emit pheromones to say “I’ve been raped” for example, and it’s as if you’ve been marked by these pheromones to indicate to the rest of the species that you’ve been raped.’* *‘That’s what really triggered me to self-sequestrate and protect myself, a drop came out of the point here and it was as if it had burned me and dripped a little like a drop of sweat, and it gave off a very strong odour, which gave me hot flashes and dizziness (…) physically it was like that, it was wet, there was really something coming out of my body at that moment*. *as if I were liquefying or as if my body was being decomposed.’* ‘*I just said to myself, I’m in danger, I’ve got to warn that I’m being attacked, I felt like it was going to paralyse me, and so, uh, I just protected myself as best I could, not knowing if I was going to survive another minute or not. (…) the intuition that someone wanted to hurt me, in fact it’s always been present in my life, given my very heavy past, it’s always been the case, it’s just that I know how to react to avoid someone hurting me, and maybe that’s why it seems a bit strange what happened, I avoided someone hurting me.’* *‘I’ve been in survival mode since I was a little girl, since I was a little baby, I was a victim of crime, so I don’t just know, I know how to do it because I have a lot of denial, but for me there’s no such thing as living serenely without survival.’* *‘I was disentangling myself from things I’d been trapped in, for me, we’re marked, those are my alchemical hypotheses*.’ *‘It was more, like, witchcraft or I sometimes felt like someone wanted to hurt me, but it was really happening, so it wasn’t really psyching me out, because it was happening in my real life, it was more that I was sensing things, the things I’ve always sensed via my intuition, my instinct. I learned a lot about alchemy, about the universe and all that, so it may have aroused jealousy, envy of theft, whatever the crime. I know there’s always jealousy, but less so because I’ve communicated about criminality, so if someone’s jealous of me they realise how weird they are, well how weird do I think it is to tell them that I’m jealous of someone who’s been raped and attempted murdered? what does that say about me? I think it deters them from being jealous. So that’s why I’ve managed to get rid of all the jealousy and meanness that’s happened to me several times in my work life.’*
2	Bullying	Suspiciousness	*‘During the episode, I always had this paranoid fear of others.’*
3	Bullying intimidation Discrimination Emotional neglect and abuse	Persecutory delusions Ideas of reference	*‘I felt like I was being attacked by them.’* *‘I began to have… to hallucinate, telling myself that maybe there are people… who know what’s happened in my life… well, that’s what was going through my mind, so I could pass someone in the street and say to myself, maybe he knows me through so-and-so, he’s heard of me.’*
7	Emotional abuse Intimidation and exclusion by a chief member of a spiritual group Death of a grandmother	Auditory-verbal hallucinations Delusion of control Spiritual ideas Somaesthetic hallucinations	*‘I hear voices insulting me and uh and uh actually since my grandmother died I often hear her voice, but not all the time, and it was from time to time and it’s mostly in nature, and it’s like they’re trying to modify my grandmother’s voice too, to make her say things she’d never say to me, and that… that she never told me.’* *‘The tone of voice was modified, the content of what she said was modified and it’s especially sometimes, especially in the morning, I hear her voice screaming, as if they were torturing her, and that’s really hard, very often.’* *‘That they’re going to kill me, that they want to harm the earth, that they want to harm human beings, who are… some kind of demon.’ ‘It’s really with this woman that the voices started.’* *‘The woman’s voice became far too powerful, and I began to feel the physiological/physical consequences, on a sensory level, of what the voices were telling me they were going to do. Like, for example, we’re going to hurt you, we’re going to take your arm apart, and then I’d feel my arm physically twist. Since I have strong spiritual beliefs, for me it was evil spirits, or her.’* *‘I felt like my body was attached by a wire, connected to her, and she was telling me that she was going to hurt me, that she was going to hurt my family, that she was going to manipulate me, that I was going to die, and that she was going to do everything she could to push my suicide spiritually, because it’s very much linked to spirituality.’*
9	Traumatic sports injuries forcing him to stop competing	Auditory-verbal hallucinations	*‘At first I thought I was convinced that it was my grandparents who were talking, who were talking to each other, and who were saying, look at what (name) has become … well, there you go, he’s destroying his life, that’s what I said to myself.’*
10	Sexual abuse before the age of 16 years old Strict religious upbringing Interpersonal tension with his parents	Auditory-verbal hallucinations Tactile distortions	*‘I had two voices, a good voice that guided me towards Islam, and a voice, the voice of reason, and the voice of the heart that guided me towards Hinduism. This bad voice pulls me down, one day the good voice disappears, only the bad voice remains, I become anxious, I feel like committing suicide, I become stressed about everything, because it tries to force me to do things I don’t want to do. It changes my beliefs, so I can’t be Muslim anymore and I become Hindu.’* *‘If someone touches me, I feel like they’re embracing me. And if someone walks behind me, I feel like they’re embracing me too.’*
11	Bullying intimidation	Suspicious ideas	*‘So, we’ve experienced a certain amount of censorship, and it’s obvious that young people realise this and discuss it among themselves, which creates a feeling of insecurity when you can see everything that’s going on among us.’*
12	Bullying Intimidation Discrimination Emotional neglect	Persecutory ideas	‘*When I was walking in the street, I felt ashamed, so I imagined that the person walking by had turned their head to look at me, when in fact they hadn’t, and I inevitably interpreted that as bad, when in fact they hadn’t even turned their head. And it turns out that even if she did turn her head, it wasn’t necessarily negative, so I interpreted everything as bad and little by little, everything that happened was directed against me in fact, everything that happened.’*
13	Abandonment and adoption Sexual abuse before the age of 16 years by his brother Bullying intimidation	Suspicious ideas	*‘I thought he was with me and I thought he was against me and so all the time there was this arguing going on, in my brain it was in fusion, eh, it was in fusion, it was arguing between knowing uh am I going in this direction because if I’m going in this direction I can trust the people who point me in this direction… or if I go in this direction or if I trust such and such a person it can uh… I can’t go in a complicated situation.’*
14	Emotional abuse Witnessing domestic violence Discrimination	Auditory-verbal hallucinations Intrapsychic hallucinations	*‘At the beginning it was insults, it was that when I was on my own, when I was with my family I didn’t have these thoughts, but when I was on my own, I started to have lots of thoughts and I’d answer myself, for example, I remember going to work, and I’d have these thoughts while I was driving. And I’d answer myself while I was driving, and that’s when I started asking myself questions.’* *‘I had bad thoughts every time, well not bad thoughts, a lot I put myself down a lot in my thoughts and that played a lot on me.’*
15	Witnessing domestic verbal violence Sexual abuse before the age of 16 years old	Auditory-verbal hallucinations with insults Tactile hallucinations	*‘I don’t even know if there’s a link or not, except that one of the voices was insulting a lot and that reminds me of my family insulting each other, but that’s all.’* *‘There were times when I felt people touching my arm.’*

### Analysis

Baseline demographic characteristics were summarised using means (for continuous data) and frequencies (for categorical data).

The qualitative part of the interviews was audio recorded, pseudonymised using a code preventing direct identification of the participants and literally transcribed. A thematic content analysis, following the approach proposed by Bardin,^16^ had been carried out using NVivo (version 14.23.2 for Windows; Lumivero, Denver, CO, USA; https://lumivero.com/products/nvivo/ ) to get as close as possible to the participants’ discourse. A vertical and transversal analysis was carried out to categorise the transcriptions into themes and subthemes. The analysis grid followed deductive and inductive approaches. Positive psychosis symptoms coding derived from a deductive analysis based on the interview structure of the CAARMS to identify known types of positive psychotic symptoms. Next, we proceeded to an inductive analysis to identify the thematic content of each code (i.e. the content of the auditory verbal hallucinations). An initial codebook was developed based on concepts from the interview guide (i.e. from existing literature, and following the structure of the CAARMS and the TALE), and those that emerged directly from the data (i.e. from two researchers’ readings (A.-A.T. and M.M.) of 17 transcripts). The two researchers, supervised by F.H. and J.H., discussed coding choices until agreement, or a new code was developed. The codebook was subsequently finalised and all transcripts were coded. Encoding was double-blind for the first interviews and compared between A.-A.T. and M.M. to limit the subjectivity of encoding, only after A.-A.T. completed the coding of all the transcripts.

From the results of the interview and the TALE administration, the prevalence of each trauma and life events before the first episode of psychosis in the sample was calculated, except for items 15 to 18 of TALE. These items explored the traumatic experience of symptoms, the distressing behaviours possibly related to symptoms, the experiences in mental health services and other contacts with health or justice services. The prevalence of multiple exposure to each trauma and life events was also calculated. The number of trauma and life events per participant and the number of trauma and life events with multiple exposure per participant was calculated. An average age at onset and age at end of exposure to each trauma type was calculated.

For the following analyses, the participants’ answers handwritten by the examiner during the TALE completion (preceding the audio-recorded interview) and the TALE results were integrated into NVivo and coded.

We used matrix coding queries in NVivo 14 to explore the association between codes of specific types of positive psychotic symptoms and codes of specific life and trauma events. Using codes for positive psychotic symptoms and codes for traumatic event histories, each participant’s file was sorted into static sets based on either the presence or absence of that specific antecedent and that specific symptom. These sets were then subjected to matrix coding queries to reveal patterns. This enabled us to highlight preferential associations between codes for each positive psychotic symptom and codes for each life and traumatic event.

We used cluster analysis in NVivo 14 with the Jaccard coefficient, to support the identification of associations between codes for trauma and life events and other codes.^17^ The use of cluster analysis in qualitative research is a documented method that supports thematic analysis and interpretation by revealing patterns in how codes co-occur across participants.^18,19^ It is complementary to thematic analysis and can help to identify emerging themes or subthemes and refine thematic analysis results. This approach also improves reproducibility of the results, and is very appropriate for exploratory studies with large textual data. Among similarity measures, the Jaccard coefficient was chosen because it is commonly used when the data is qualitative and binary, such as the presence or absence of a feature. The Jaccard similarity coefficient accounts for shared occurrences of codes without inflating similarity owing to non-occurrences. The Jaccard similarity coefficient had been used to evaluate the coding similarity, i.e. it measured the proportion of participants having these two codes relative to the total number of other participants having one of these codes. The Jaccard coefficient ranges from no similarity (0) to full similarity between codes (1). According to the rules from Evans correlations, we considered correlation between 0.40 and 0.59 to be moderate, between 0.60 and 0.79 to be strong and between 0.80 and 1.0 to be very strong.^20^

## Results

### Description of the sample

The sample consisted of 17 individuals currently enrolled in the PEP’S FEP programme (see demographic characteristics of the sample in Table 2). Of them, nine participants were recruited from the out-patient unit and eight participants were recruited from the specialised in-patient unit for FEP. A.-A.T. knew eight of the 17 participants before the study start, from residency in the FEP programme; she also had a work history with the clinical team engaged with all the participants. This situation increased confidence between patient and interviewer and eased the interview process. Also, the use of a systematic semi-structured interview allowed reproducibility. Of the nine participants recruited from the out-patient unit, eight of them were admitted to hospitals outside of the specialised FEP programme in general psychiatric units during their first psychotic episode, and one had not been admitted to hospital. Only two participants refused to participate in the study. These two participants did not wish to have any additional interviews beyond those scheduled as part of their usual care.

**Table 2:** Characteristics of the participants interviewed (*N* = 17)

Characteristics	*n*	%
Male	10	58.8
Age in years		
18–21	4	23.5
22–27	12	70.58
≥28	1	5.8
Length of time in programme		
<1 month	4	23.5
1–6 months	3	17.6
>6 months	10	58.8
Years of education		
7–16 years	6	35.3
>16 years	11	64.7
Diagnosis		
Brief psychotic disorder	2	11.7
Schizophreniform disorder	6	35.3
Schizophrenia	5	29.4
Bipolar disorder with psychotic features	2	11.7
Schizoaffective disorder	2	11.7
Medical and psychiatric history		
Obstetrical complications	3	17.6
Congenital disorder	2	10.6
Depressive episode	4	23.5
Anxiety disorder	3	17.6
Borderline personality disorder and suicide attempt	1	5.8
Dyslexia	1	5.8
Attention-deficit hyperactivity disorder	2	10.6
Family medical and psychiatric history		
Bipolar disorder or schizophrenia	4	23.5
Suicide	1	5.8
Epilepsy	1	5.8
Cancer	1	5.8
Ocular disorder	1	5.8
Substance misuse	6	35.3
Alcohol	4	23.5
Cannabis	4	23.5
Cocaine	1	5.8
Psychedelic	1	5.8
Minority ethnic group	10	58.8
First- or second-generation migrants	6	35.3

### Results of the TALE questionnaire

Sixteen out of 17 participants reported past life and trauma events (see Table 3). All but one participant reported multiple trauma and life events. One participant identified the first episode of psychosis as the only trauma event in their life. The average number of trauma and life events before FEP was six. The average number of trauma and life events with multiple exposure before FEP was four. Most participants identified trauma and life events still affecting them (with a current emotional impact), in particular sexual abuse before the age of 16 years old, witnessing domestic violence, bullying, psychosis, emotional neglect, accidental events, temporary separation from a close relative, migration and emotional abuse.

**Table 3:** Trauma and life events cross-referenced from the Trauma and Life Experiences Checklist, interview and medical file

Trauma and Life Experiences Checklist item	Frequency, *n* (%)	Repeated exposure, *n* (%)	Average age at onset	Average age at end
15. Psychosis (symptoms)	14 (82.3)	11 (64.7)	21.6	22.4
16. Psychosis (unusual behaviours)	11 (64.7)	9 (52.9)	20.6	22.3
4. Unexpected move or loss of home	11 (64.7)	5 (29.4)	11.3	13.5
5. Bullying	9 (52.9)	9 (52.4)	11	14.3
17. Psychosis (treatment/hospital admission)	9 (52.9)	6 (35.3)	20.25	22.3
7. Emotional abuse	8 (47)	8 (47)	9.2	19.1
8. Emotional neglect	8 (47)	6 (37.5)	5	17.25
3. Period of separation from caregiver	8 (47)	3 (17.6)	7	11.6
6. Discrimination	7 (41.1)	6 (35.3)	15.5	17.8
9. Witnessing violence at home	7 (41.1)	6 (35.3)	5.5	13.3
13. Childhood sexual abuse	6 (35.3)	4 (23.5)	10.25	12
10. Violence outside of home	6 (35.3)	3 (17.6)	16	19.3
5. Permanent separation or loss	5 (29.4)	2 (11.7)	10.3	13
8. Physical abuse	5 (29.4)	2 (11.7)	10.3	13
19. Accidents and illnesses	3 (17.6)	3 (17.6)	10.5	16
12. Physical neglect	3 (17.6)	3 (17.6)	10.5	16
1. Exposure to war and civil unrest	2 (11.7)	2 (11.7)	12.5	17
14. Unwanted sexual experiences in adulthood	2 (11.7)	1 (5.8)	19	20
18. Other experiences with health/justice service	2 (11.7)	0	23	

### Results of the thematic content analysis

After conducting thematic content analysis, five major themes were identified: theme 1, types of positive psychotic symptoms; theme 2, context of onset of positive psychotic symptoms and precipitating factors of positive psychotic symptoms; theme 3, experience of positive psychotic symptoms; theme 4, thematic association between trauma and psychosis; and theme 5, causal or mediating factors of positive psychotic symptoms.

#### Types of positive psychotic symptoms

All participants described positive psychotic symptoms during the qualitative interview: perceptual anomalies, delusions and non-bizarre ideas, unusual thought content and disorganisation (see Box 1 for the detail of positive psychotic symptoms and the thematic content of each code).

Box 1 Theme 1: symptoms with subthemes and codes detail and number of individuals among the 17 participants interviewed for each code

Perceptual anomalies (13/17)

- Auditory-verbal hallucinations: content: control, body/bones, death, imperfection/failure, exclusion, injunction to arm oneself/threatens, insults/derogatory remarks, spiritual (identity of voices, relation with the person, powerful voices)
- Somaesthetic anomalies: heat, pain/twisting/decomposition/strangulation, electricity, sore throat/dryness, headaches, muscular pain
- Tactile hallucinations: drop of sweat/wet, liquefaction, gripped/touched/embrace, burn, pressure on orifices, insect
- Visual hallucinations: creatures/monsters, people/shadows, animals, beads of light/spiritual/symbols/cathedral vault, fire/blood/violence/red, particles
- Auditory hallucinations: clock noise, tik tak noise, neighbour noise, screams, hubbub
- Auditive distortions: auditory hyper-reactivity, greater sensitivity
- Visual distortions: visual hyper-responsiveness, improved visual acuity, object movement, deformation
- Gustative distortions: taste of paper, blood, limestone, dog food
- Olfactory hallucinations: failure to recognise the scent of one’s own laundry detergent or perfume, a more sensitive sense of smell
- Tactile distortions: embracing sensation, tactile hypersensitivity

Delusions and non-bizarre ideas (16/17)

- Persecutory and suspicious delusions: tracked, monitored, spied on, negatively judged, envied, life-threatened, ideas of distrust
- Grandiose delusions and ideas: megalomaniac ideas, ideas of controlling other people, ideas of liberation, societal ideas
- Mystic or esoteric delusions: bewitchment, ideas of incarnation, esoteric ideas, religious ideas, ideas of creating and end of the world
- Somatic delusions: pheromone release ideas, ideas of liquefaction, ideas of physical intrusion, ideas of being mute or deaf, ideas of having hot flashes, ideas about being stained, ideas of having a physical problem
- Nihilistic ideas: feeling of dying, sensation of near death, feeling of being dead
- Delusion of control: ideas of being tied down, tortured and controlled by a malefic being, ideas of being controlled by a higher being, ideas of being controlled by voices
- Other non-bizarre ideas: culpability ideas, ideas of infestation, nature-related ideas

Unusual thought content (12/17)

- Delusions of reference
- Perplexity: strangeness, wrong wave, astonishment, doubt, questioning
- Imposed thoughts and feelings: thoughts in the wrong direction, feeling imposed
- Intrapsychic hallucinations: self-deprecating
- Delusions of thought broadcasting: remarks about others, judgement
- Delusions of thought reading: disease, pregnancy

Disorganisation (12/17)

- Disorganised behaviour: abnormal clothing, screams, pathological buying, research on a deceased person, mess/damage/tearing documents, laying down in the street, boiling and cold showers/cold water, dark clothing, pathological fandom
- Disorganised speech: incoherent, illogical speech, unclear association
- Soliloquy: screams, talk, mimetic
- Inappropriate affect: unprovoked laughter, grimace

Negative symptoms (5/17)

Post-traumatic symptoms (7/17)

- Nightmares, flashback, revival, avoidance, hypervigilance
- Post-traumatic symptoms of positive psychotic symptoms

Other psychopathology (17/17)

- Depressive symptoms: depressed mood, anhedonia, ideas of incurability
- Sleep disturbance: insomnia, nychthemeral rhythm inversion
- Reduced or difficult communication
- Fatigue
- Suicidal ideas
- Tachypsychia
- Organisational difficulties
- Increased energy
- Dissociation, depersonalisation
- Incuria
- Disturbance of appetite
- Obsession and compulsion

#### The context of onset and precipitating factors of positive psychotic symptoms

All but one participant identified a specific context of onset of the FEP.

Most participants described positive psychotic symptoms as emerging in the context of trauma and life events.

Some participants described positive psychotic symptoms following post-traumatic symptoms.

The other contexts identified were travel overstimulation or climate adaptation, toxic consumption or weaning, socioeconomic or professional concern and overwork.

#### Experience of positive psychotic symptoms from the perspective of participants with FEP, including emotional and distressing experiences

The participants’ experience could be gathered into four subthemes: the emotions felt, the behaviours adopted by participants when experiencing positive psychotic symptoms, and the cognitive and metacognitive perturbations described by participants. Most participants described positive psychotic symptoms as a distressing experience, with fear and anxiety (see emotional responses in Box 2). They described cognitive bias, memory perturbations and metacognitive perturbations, as well as dysfunctional behavioural responses that probably maintain the distressing experience of positive psychotic symptoms (see codes, prevalence and quotations in Box 2, Theme 3: experience of positive psychotic symptoms, codes with quotes).

Box 2 Theme 3: experience of positive psychotic symptoms, codes with quotes

Emotional response (17/17)

- Fear or anxiety:
  - *‘I was afraid of being hurt*’ (participant 2)
  - *‘I was afraid to die*’ (participant 4)
  - *‘I put myself in a state of total panic’* (participant 7)
  - *‘Bad voices that put me down’, ‘I was afraid of not knowing how to defend myself… of not being safe’* (participant 14)
  - *‘I’d get really nervous’* (participant 15)
- Anger:
  - *‘Anger in fact, a lot of anger and no more control, no more control over myself in fact, at that moment I was elsewhere, I was no longer myself in fact, I was no longer how to say uh… I was another person in fact’* (participant 16)
- Sadness:
  - *‘I didn’t feel well, I mean I wasn’t happy, and in fact I felt mostly, I don’t know if that’s the right word, overwhelmed’* (participant 15)
- Association of fear, anger and sadness:
  - *‘Fear, terror, stress, anxiety, deep sadness and a feeling of helplessness that you’ve tried everything you can think of’* (participant 7)
  - *‘It’s stressful. So inevitably because you don’t understand, I’d say it was a surplus of stress in terms of emotion. It can also lead to a certain amount of sadness, because you don’t understand, and it’s always unpleasant to see sudden changes that you can’t control, so I’d say there was a certain amount of sadness, and I sometimes cried myself to sleep’* (participant 11)
  - *‘Well, it affected me… it made me sad, after all, I very rarely cry, I’m very sensitive, but it’s very rarely that I cry, but that day, it wasn’t fear, it was… it was the injustice that I… I felt. In fact, uh, I felt… well… I just felt misunderstood’* (participant 13)
- Excitement, joy:
  - *‘He’d tell me to look for the loophole, and it’s something that’s marked me, almost exalted me. There were all the famous singers, that’s in my hallucinations I think, all the famous singers who sang for me, but against me, who challenged me to a duel, a battle, and I had to sing, dance, and I was actually having fun. The same thing happened when, in my head, I was contacting the secret service (laughs) and I was thinking, okay you’re being very careful, I think that in real life my parents, looking at me from a distance, the doctors, must have thought, she’s talking really loudly, I don’t know (laughs) but yeah, it was upsetting, but it made me feel good, it’s weird, it really made me feel so good’* (participant 2)
- Blunted affect, difficulty to specify emotion:
  - *‘It’s as if I had no emotions at all since, I kept everything inside me I’d told my parents about this malaise but as soon as I saw that … they didn’t show up to uh … for my health to improve uh … I kept everything inside me’* (participant 17)
- Shame:
  - *‘It was like I was dying, I was ashamed of myself, and I was at my lowest ebb, that’s all…. Shame, embarrassment’* (participant 12)

Associated perturbations of cognitions (13/17)

- Memory perturbation: amnesia, association, disconnection, selective memory, over general memory
- Cognitive bias: attribution bias, disruption of attentional control, salience bias, interpretation bias, emotional reasoning, magical thinking, arbitrary inference

Associated metacognitions (17/17)

- Cognition about one’s functioning: ‘*I feel like my mind has split into several pieces’* (participant 15)
- Cognitions about the liberating virtue of the symptoms: *‘By living through this event, it allowed me to free myself from my trauma, and maybe my brain would react better next time. (…) so when we suffer a crime, we emit pheromones to say “I’ve been raped”, for example, and we’re marked by these pheromones to indicate to the rest of the species that we’ve been raped, so to free ourselves, I’ve found nothing other than to repeat the crime in reverse on ourselves, to be the rapist and the raped, so that cancels each other out, and as a result I noticed that there were odour emanations, but very unpleasant ones for example, that’s pretty horrible to say, but I self-polished to try and free myself from it. And once I’d done that, I don’t even have words to describe how I felt after I’d done that, because I felt so good. But on the other hand, it was a horrible act and I made a slightly protective denial of it, to tell myself no, I wasn’t raping myself, but rather no, I was taking pleasure with myself, rather than telling myself that I was raping myself to free myself, I told myself more, I made love to myself, telling myself that it made me feel much better afterwards, so it was an act of love, rather than a criminal act’* (participant 1)
- Cognitions about self-representations
  - Cognition about identity and one’s existence: ‘*In fact, I didn’t understand myself, I knew who I was but deep down I was nothing, in the end I was just a little speck of dust on the planet like everyone else, and the fact that I said to myself, I’m almost nothing’* (participant 2)
  - Negative belief about the self*: ‘I didn’t particularly like myself’* (participant 2)
  - Body image: ‘*When you’re walking down the street and you imagine you’ve got a stain etc., and I’m not talking about a stain like that, you always imagine the worst, so you can’t walk with your head held high’* (participant 12)
  - Increased sense of self-efficacity and accomplishment: ‘*I’ve even regained my self-confidence, I’ve always had little self-confidence because of all this, and I said to myself, you can still do things on your own!’* (participant 1)
  - Decreased sense of self-efficacity and accomplishment, sense of failure: *‘The things I was supposed to deal with, I just couldn’t deal with them anymore’* (participant 3)
  - Lack of understanding/feeling lost: ‘*I would say distraught, lost’* (participant 7); *‘I didn’t understand’* (participant 10); *‘I understood nothing, I was lost’* (participant 14)
  - Discrepancy between the self-perceived by others, the desired self or the actual self: *‘I compare myself to people and if I compare myself and I don’t like it and it doesn’t suit me, well, I’ll brood about it’* (participant 9)

Behavioural response (16/17)

- Behavioural avoidance:
  - Withdrawal to home: *‘I just wanted to isolate myself and that’s why I didn’t go out for the 6 months in 2022 from September to December’* (participant 12); *‘I only went out to eat and to the toilet and shower. I stayed all day with a fan and in the dark’* (participant 17); *‘I didn’t leave my house anymore’* (participant 9)
  - Social withdrawal: ‘*I had begun to cut ties with everyone’* (participant 14); *‘In those moments I no longer saw the other people’* (participant 15)
- Repression/denial: ‘*There is nothing that bothers me’* (participant 17); ‘*I personally don’t mind. I’m a person, that’s all’* (participant 8)
- Thought suppression: ‘*My family was devastated by it, even my cousins and uncles, it went everywhere, everyone knew it. And I wasn’t proud of it. I think I’ve gone through a phase where I don’t want to think about it anymore and I’ve forgotten a lot of stuff’* (participant 14)
- Distress endurance: ‘*I was just thinking, I’ll take it as it comes and see where it takes us’* (participant 1)
- Ruminating and questioning: ‘*It works on me; it works on my mind’* (participant 3); *‘I began to wonder about things, my analyses began’* (participant 6); *‘It was the beginning of a long reflection’* (participant 11); *‘I was thinking of ten thousand things, ten thousand solutions, um, treatments, what I could do, could I see energy therapists, were there plants that could help me with that to calm my anxieties’* (participant 7); *‘I could spend hours thinking about questions that are on my mind’* (participant 12)
- Distraction/suppression:
  - Toxic consumption*: ‘I used to drink alcohol to fall asleep’* (participant 3); *‘I used drugs at home… Saturdays and Sundays, as soon as I had days off, I’d get high on … coke or alcohol’* (participant 9)
  - Overinvestment in studies or work: ‘*I went on with my life, working every day of the week’, ‘I was congratulated at work’* (participant 9); *‘I was a bit overinvested in my studies’* (participant 15)
  - Compulsion: ‘*checking, OCD [obsessive–compulsive disorder]’* (participant 12); *‘I had a kind of hypnotic crisis, I don’t know if you can say it, where I couldn’t get out, I was like, well, it’s also buttons that I’ve always itched since I was little, when I do it, I can’t stop, it’s horrible, and for me I call it a hypnotic moment because I’m hypnotised at the time and I can’t get rid of it, and it relieves me because it allows me to exteriorise’* (participant 2)
- Help-seeking behaviours: ‘*I called my friend’* (participant 2); *‘I made sure to call ahead’* (participant 1)
- Activation and communication with others: ‘*I wrote my book and all that’* (participant 1); *‘I wrote a sort of letter to the teacher, which is a bit strange, but I’d have to find it in my file, I think it’s there, where I explained a bit what I thought about the world’* (participant 5); *‘I wrote a letter’* (participant 6)

Factors influencing symptoms

- Toxic consumption
- Sleep
- Anxiety, stress
- Behaviours adopted
- Loneliness
- No factors identified

#### Thematic associations between specific positive psychotic symptoms and specific life and trauma events

Numerous direct and indirect thematic associations were identified, but only those made by the participants were reported (see Table 1, Theme 4: thematic associations between specific positive psychotic symptoms and specific life and trauma events with quotes from the perspective of participants).

#### Causal or mediating factors of positive psychotic symptoms

The majority of participants developed causal theories about the first episode of psychosis and positive psychotic symptoms. Only three participants did not develop causal theories about the first episode of psychosis, and all of them had negative symptoms in the foreground at the time of the interview.

Most participants had theories including life and trauma events as causal factors of positive psychotic symptoms (see Box 3. Theme 5: participants’ causal theories of positive psychotic symptoms, with number and quotes). Of them, one participant described the positive psychotic symptoms to avoid exposure to ongoing trauma events. Three participants mentioned their life and trauma events, but did not know if they were causal factors. One participant who migrated to France to study and work, identified the recent separation from his family as traumatic, but made no connection with the first episode of psychosis. Four participants described psychological processes as mediating factors: negative belief about others because of trauma events, repetitive questioning or attribution bias.

Box 3 Theme 5: Participants’ causal theories of positive psychotic symptoms with number and quotes

**Table:** 

Causal theories implicating life and trauma events (11/17)
**Anxiety as a mediating factor between life and trauma events and positive psychotic symptoms** *‘But in the end, with hindsight, I realise that there was a lot of anguish accumulated over years and years and years, so in the end it didn’t come from nowhere.’* (participant 12) ‘*I think the concern that people close to me may have had for me and during my youth, given my life path, and that concern and stress, I think when you’re young you feel it and it has an impact.’* (participant 11)
**A cumulation of past and present life and trauma events** Participant 3 presented paranoid delusion and visual hallucination, and described his first-episode psychosis as triggered by conflictual relationship and a history of emotional neglect, emotional abuse and physical abuse by his father.
**Association between biological factors, trauma events and self-confidence** *‘I think it’s both, I think there’s family history that made the disorder develop and that made me have these hallucinations. I don’t know, but there’s also something that’s always made me feel that I never really had any self-confidence when I was little, and that’s the fact that I was very much sheltered by my parents when I was little, and by my mother in particular. Uh… and that’s something I blamed her for recently, I said to myself again, but it’s because of that, that I don’t have confidence in myself etc. Today, it’s maybe partly, but it’s not only because of myself…all the time apologising, saying sorry or really feeling inferior to others.’* (participant 2)
**Traumatic intrusion, memory, emotional reasoning, paranoid delusion and hallucinations** Participant 1 identified childhood abuse as a causal factor and she discussed the link between trauma events, reviviscence, paranoid delusion and hallucinations: *‘For me there is indeed a psychotic episode, but there is also, the post-traumatic shock that was already in me, and there is always another component that seems strange to me, which I call witchcraft or something else because I can’t explain it. I think that in fact it’s the fact of feeling in danger that has reemerged everything I’ve ever experienced in danger, because I was precisely in a phase of liberation from my past, and so the link is more the memory. I don’t know… memories I might have had that came back that day, and suddenly, and maybe that’s what triggered an episode.’*
**Emotional neglect, lack of self-affirmation and megalomaniac delusions** Participant 6 presented with megalomaniac ideations and persecutory delusions. ‘*In fact, my difficulties can be traced back to my mother’s love. All I wanted in my life was my mother’s support. In fact, every time I wanted to assert myself, I was afraid to do so. I wanted her support just, but I couldn’t have her support right now, but I couldn’t have her support right now, because she was busy taking care of us, since my dad was sick, so me in general, so, I wanted her support to feel stronger, but since every time I showed my results, even if I was first, she trivialised it she didn’t make me feel that I had done well, whereas I was always fighting to prove to her that I could and that I was better. I missed my mother, and the lack of her also prevented me from expressing everything I felt, so it’s like closing a valve, because if you don’t squeeze enough, it overflows.’* (participant 6)
**Sexual abuse, identity dissociation and delusions of incarnation** *‘My aggression weakened me to the point that we broke in two to live through it, so it’s hard, afterwards it means that the brain preferred to break in two to… to limit the damage. One could imagine that it attacks the brain, the brain suffers too much, so it’s obliged to break in two to uh divide the load in fact, and knowing that after dividing the load, there will always be one who will be more affected than the other because there’s now one who will have to protect the other in fact.’* (participant 10)
**A cumulation of disruptive attachment, emotional neglect and bullying** Participant 12 presented with paranoid delusion and obsessive-compulsive symptoms and identified the history of bullying in adolescence and of family instability as causal factors of his episode: *‘Yes, this feeling of insecurity, of seeing and thinking that people wanted to hurt me, etc. yes, the harassment I experienced at the time had an impact, and still has an impact now. And an imbalance in my growth, whether at the family level, with my father. The fact that my parents split up, so I was living with my mother, and already we all need one to have two parents, to have a father, then my father, he was going to take money, he was over-indebted etc., uh at one point he didn’t have a home anymore, so he was really in a difficult situation, I couldn’t really count on him. And I never managed to have a father-son relationship with him, and the fact that I was only with my mother, that I was imprisoned, that also made me anxious, I couldn’t manage to emancipate myself.’* (participant 12)
**Witnessing violence at home, auditory-verbal hallucinations and soliloquy** *‘I was living with my grandparents and they were always yelling at each other all the time, but I really couldn’t stand to hear him yell like that anymore, even several times when I was in my grandfather’s car with my grandmother and my uncle, that at every stop I wanted to open the door and just walk, just get out of the car even though it was still moving. And, uh… so he’d been fighting a few times and in fact, uh, I remember very well, I don’t know, but while he was fighting, I was in front of my mirror looking at myself, and I was talking to myself, and I think that, at that moment, I started hearing things, and it wasn’t pleasant, and even there were voices saying that they were called like me, etc. I have the impression that my mind split into several pieces.’* *‘I don’t know if seeing my family fighting all the time, if it helped or not, I don’t know at all, maybe it did, in fact, I don’t know how the brain really works, or the traumas, etc., if it had repercussions for the rest of my life, I don’t know if it had repercussions for my later life, maybe what happened when I was younger in terms of fights, even up to the terminal, fights or people in the family insulting each other, etc., I don’t know if it had repercussions on what I had, but what I can say is that when I was in second grade, when it happened to me, where I started to hear voices was when my family was fighting, I don’t know if I just wanted to uh be in my bubble and not hear anymore, I don’t know.’* (participant 15)
**Homelessness, paranoid delusion and megalomaniac ideas** Participant 13 had been abandoned by his mother at the age of 3.5 years old, and he developed first-episode psychosis a few years after being kicked out of home at the age of 21 years and not supported by his college, so that he became homeless. He identified an event of his belongings being rifled through when he was homeless as a trigger of his paranoid delusion: ‘*I think there’s one thing in relation to the mistrust I had too, this fear of always being followed or whatever, I think it’s because… while I was out I was out but before I left for Paris etc., when I was still working uh… at that time uh… I came back once I was a bit preoccupied in my mind saying to myself, my things, I hope nobody’s going to take them uh… and at one point I went back to the place where I’d put my things and I saw that everything had been turned upside down, I said to myself ok already the hiding place is visible, it just so happens that, as it was a small town, it just so happens that it’s someone who knows me so what’s more it’s someone who knows me and who did this and who sees. who did this to me, so it was a shot in the back, a shot in the back. And… that’s when I started to get suspicious and then I… at work even at work I thought they didn’t want me… at work.’* (participant 13)
**Causal theory: Family history of psychiatric disease (4/17)**
**Causal theory: toxic consumption (3/17)**
**Causal theory: physical problem (2/17)**
**Mediating factors: personality traits or anxiety (3/17)**
**Mediating or causal factor: context of onset (4/17)**: sleep disturbances, a sense of failure or an inability to adapt, a conflict of value, unmet needs or abandonment.

Participants were asked their opinions on a narrative interview discussing the link between their positive psychotic symptoms and trauma and life events.

The majority of participants identified the interview as relevant and helpful. The arguments put forward were to understand psychosis considering their life history, because the two of them fit together, to integrate psychosis and construct a narrative, to accept the disorder and to turn the experience into something positive. One of them specified that it is more helpful shortly after the first episode, because after they did not appreciate talking more about it. One of them found it helpful but could not tell why.

One participant identified a link with the context of onset of the first episode of psychosis and explained that they did not need help to develop it.

Four participants did not think there was a link between trauma and psychosis, and thus did not see the relevance of such an interview.

### Exploration of the association between specific positive symptoms and specific trauma and life events drawn from the matrix coding queries

For this phase, the participants’ answers handwritten by the examiner during the TALE and the TALE results were integrated into NVivo and coded.

Exploratory associations between specific positive psychotic symptoms and specific trauma and life events have been drawn from the matrix coding queries (see Table 4). The main associations were:

- Tactile perceptual anomalies, bewitchment or incarnation ideas, thoughts/images/acts imposed on self, auditory-verbal hallucinations, combination of megalomaniac and persecutory ideations and sexual abuse before 16 years old. Three participants had all of these symptoms combined; all of these participants reported sexual abuse. The association between all of these symptoms was only found in patients reporting sexual abuse;
- Combination of ideas of grandiosity and control and ideas of being prey to others, disorganised behaviour and permanent loss of caregiver and physical neglect;
- Soliloquy and witnessing domestic violence;
- Persecutory ideas and bullying, intimidation and emotional neglect.

Exploratory associations between trauma and life events codes, positive psychotic symptoms codes, and other codes (emotion, cognitive and metacognitive, context of onset, thematic association, causal theories) have been made with cluster analysis (see Table 5). Only trauma and life codes present in at least three participants are shown. We found that intimidation or bullying was correlated with megalomaniac delusion; emotional abuse with disorganised behaviour; emotional neglect with ideas of reference, grandiose delusions, suspiciousness and paranoid delusions; childhood sexual abuse with tactile hallucinations, mystic or esoteric delusions, ideas of incarnation, auditory verbal hallucinations, megalomaniac delusions and delusion of control; and witnessing domestic violence with soliloquy.

**Table 4:** Other exploratory associations between specific positive psychotic symptoms and specific trauma and life events drawn from the matrix coding queries of the qualitative analysis

Subtype of positive psychotic symptoms (*n*) or combination of subtypes of positive symptoms	Type of trauma and life events preferentially associated with this subtype of positive psychotic symptoms	Prevalence of this type of trauma and life events among participants having this positive psychotic symptom	Prevalence of this type of trauma and life events among participants not having this symptom
Tactile anomalies (6/17)	Sexual abuse before the age of 16 years old	5/6	1/11
Mystic or esoteric ideations (6/17)	Sexual abuse before the age of 16 years old	5/6	1/11
Thoughts/images/acts imposed on self (5/17)	Sexual abuse before the age of 16 years old	5/5	0/11
Auditory-verbal hallucinations (6/17)	Sexual abuse before the age of 16 years old	5/6	1/11
Combination of megalomaniac and persecutory ideations (6/17)	Sexual abuse before the age of 16 years old	5/6	1/11
Olfactory hallucinations (2/17)	Sexual abuse before the age of 16 years old	2/3	4/14
	Physical abuse	3/3	2/14
	Emotional abuse	3/3	4/14
Combination of grandiose delusion with ideas of control of others, delusion of being prey to others and behaviour disorganisation	Permanent loss of caregiver	2/2	1/15
	Physical neglect	2/2	3/15
	Discrimination	2/2	3/15
	Witnessing violence at home	2/2	6/15
	Emotional neglect	2/2	6/15
Ideas of being envied by others	Accidents and illnesses	2/2	2/15
Persecutory delusions (11/17)	Discrimination	4/11	1/6
	Bullying and intimidation	7/11	2/6
	Emotional neglect	6/11	2/5
	Permanent loss of caregiver	3/11	0/5
Soliloquy (5/17)	Witnessing violence at home	5/5	3/12

**Table 5:** Cluster analysis to explore similarity and dissimilarity between codes of trauma and life events, and other codes (with Jaccard coefficient superior to 0.5 indicated)

Trauma and life events (number of participants)	Other trauma and life events or context codes	Emotional response, cognitions, behavioural response, metacognitions codes	Symptoms, psychotic symptoms codes	Codes of causal theories, evaluation of the interview and association
TALE psychosis (11/17)	Social and circumstantial factors (0.82) Socioeconomic or work concern or change (0.5) TALE intimidation or bullying (0.5)	Fear and anxiety (0.87) Lack of understanding, feeling lost (0.6) Memory perturbation (0.6) Cognitive bias (0.58)	Paranoid delusions (0.68) Suspiciousness (0.6) Auditory hallucination (0.56) Disorganised behaviour (0.53)	Causal theories association between positive psychotic symptoms and life and trauma events (0.58) Interview relevant and helpful (0.58)
TALE unexpected move or loss of home (11/17)	TALE intimidation or bullying (0.82) TALE psychosis (0.62) Context social and circumstantial factors (0.58) TALE temporary separation of caregiver (0.54)	Memory perturbations (0.54) Cognitive bias (0.53)		Causal theories association between positive psychotic symptoms and life and trauma events (0.64)
TALE intimidation bullying (9/17)	Unexpected move of home (0.75) Context social and circumstantial factor (0.56) Emotional neglect (0.5) Emotional abuse (0.5)	Emotion: sadness (0.5) Memory perturbations (0.5) Sleep disturbance (0.5) Cognitive bias: arbitrary bias (0.5) Metacognition: cognitions about self-representation: lack of understanding and feeling lost (0.5) Perturbation of thoughts (0.53) Cognitive bias (0.61) Perturbation of attentional control (0.5) Help-seeking behaviour (0.63)	Perceptual anomalies (0.6) Delusions (0.58) Positive psychotic symptoms (0.58) Unusual thoughts content (0.57) Grandiose delusion megalomaniac ideas (0.5) Disorganisation (0.5) General psychopathology (0.58)	Causal theories association between positive psychotic symptoms and life and trauma events (0.61) Interview relevant and helpful (0.6)
TALE emotional abuse (8/17)	Context: Socioeconomic or work concern or change (0.54) Temporary separation of caregiver (0.54) Context: Social and circumstantial factors (0.5) Bullying and intimidation (0.5) Witnessing domestic violence (0.5)	Emotion sadness (0.55) Reduced sense of efficacy and self-efficacy, lack of understanding and feeling lost (0.54) Misunderstood (0.5) Help-seeking behaviour (0.63)	Disorganisation (0.53) Disorganised behaviour (0.5) Fatigue (0.62) Post-traumatic symptoms (0.5)	Causal theories association between positive psychotic symptoms and life and trauma events (0.54) Causal theories link with the context (0.5)
TALE emotional neglect (8/17)	Context: Socioeconomic or professional changes or concerns (0.7) Context: Social and circumstantial factors (0.5) Bullying and intimidation (0.5) Witnessing violence at home (0.5)	Reduced sense of efficacy and self-efficacy, lack of understanding and feeling lost (0.54) Fear and anxiety (0.54)	Ideas of reference (0.55) Grandiose delusions (0.54) Suspiciousness (0.54) Paranoid delusions (0.53) Unusual thought content (0.53) Absence of a symptom (0.5)	Causal theories including trauma and life events (0.67) Interview relevant and helpful (0.53)
TALE discrimination (7/17)	Threatening contact with the police (0.5)	Anger (0.66) Endangerment (0.57) Thoughts acceleration (0.5) Dissociation (0.5) Fatigue (0.5)	Visual hallucinations, creatures or monsters (0.5) Auditory-verbal hallucinations, powerful voices (0.5) Tactile hallucinations (0.5)	Interview relevant existence of a link between trauma and positive psychotic symptoms (0.5)
TALE witnessing domestic violence (7/17)	Context: Socioeconomic or professional changes or concerns (0.6) Emotional neglect (0.5) Emotional abuse (0.5) Permanent loss of caregiver (0.5)	Lack of understanding and feeling lost (0.6) Endangerment (0.55) Altered cognition about self (0.5) Altered behaviour (0.5) Fatigue exhaustion (0.5) Fear and anxiety (0.5)	Soliloquy (0.57)	Causal theories trauma and life events (0.5) Interview relevant and helpful (0.5)
Childhood sexual abuse (6/17)		Emotion anger (0.5) Memory perturbations (0.5) Mental imagery (0.5) Help-seeking behaviour (0.55)	Tactile hallucinations (0.83) Mystic or esoteric delusions (0.71) Mystic or esoteric delusions ideas of incarnation bewitchment (0.66) Auditory verbal hallucinations content (0.62) Delusion of control (0.5) Hallucinations of torsion, decomposition, strangulation (0.5) Grandiose delusion megalomaniac (0.55) Grandiose delusions (0.5) Injunction to hurt oneself, threat in auditory-verbal hallucination (0.5) Interior auditory-verbal hallucination (0.5) Visual hallucinations with writing or blood (0.5) Somatic or somesthesic hallucinations (0.5) Somatic distorions (0.5)	Causal theories including trauma and life events (0.54)
TALE physical abuse by close ones (5/17)	Context: Sociocultural factors (0.5)	Increased energy (0.5) Consequences of behaviour: endangerment (0.5) Other symptoms (0.5)	Auditory distortions (0.5)	Association between trauma and life events and contextual factors (0.5) Link made with sociocultural elements (0.5)
TALE permanent loss of caregiver (5/17)	Context: Socioeconomic concern or change (0.55)	Behavioural response: activation, communication (0.5)	Mind reading (0.5)	Misunderstood during care (0.5) Link made with sociocultural elements (0.5)
TALE accident and illness (4/17)		Behavioural response: Hyper-investment in school or work (0.67) Suicide attempt (0.66)	Ideas of being envied by others (0.66) Hallucination link with traumatic events (0.5) Auditory-verbal hallucinations, Injunction to hurt oneself (0.5)	Causal link between hallucination and trauma events (0.5)
TALE physical neglect (3/17)	Context: Toxic withdrawal (0.5) Context: Social and circumstantial factors: Professional tension (0.5)	Guilt (0.5) Post-traumatic stress symptoms: Nightmares (0.5) Altered behaviour seeking to defend oneself or attack with a knife (0.5) Traumatic stimuli (0.5) Cognitive bias: Attribution bias (0.5)	Auditory hallucination, neighbour noise (0.5) Guilt ideations (0.5) Ideations of infestation or degradation (0.5) Incuria (0.5)	Causal theory: attribution bias as a mediating factor (0.5) Causal theory: association between life and trauma events and positive psychotic symptoms: stressful circumstantial factors and emotional neglect (0.5) Causal link with emotional abuse (0.5) Causal theory: link made with introversion (0.5) Interview relevant and helpful (0.5)

TALE, Trauma and Life Experiences Checklist.

## Discussion

This study investigated the experience of positive psychotic symptoms and the association between positive psychotic symptoms, life and trauma events, from the perspective of people who have experienced a first episode of psychosis.

To our knowledge, this is the first study to explore the experience of FEP among patients in France. Our qualitative findings give important insights into the experience of FEP and individual beliefs about the causes of their distress. We asked patients for their opinion on a possible association with their life and trauma events and the relevance of such interviews in routine care. Our study also gives exploratory data on the period of exposure to life and trauma events in this population, and on the association between specific positive psychotic symptoms and specific trauma and life events. Most of the participants were exposed to cumulative trauma.

Our study revealed that the experience of positive psychotic symptoms was associated with emotional distress for most participants; some subtypes of positive psychotic symptoms were preferentially associated with trauma events; there were thematic association between trauma events and the symptoms, and, for some participants, positive psychotic symptoms were associated with (or preceded) dissociation and post-traumatic symptoms.

### Association between specific types of positive psychotic symptoms and trauma and life events, and characteristics of positive psychotic symptoms

The association found between tactile anomalies, mystic or esoteric ideations, thoughts/images/acts imposed on self, auditory verbal hallucinations, combination of megalomaniac and persecutory ideations, and sexual abuse before 16 years of age is consistent with some other studies.^21,22^

Ideas of controlling others and being prey to others and behavioural disorganisation were associated with permanent loss of caregiver, physical neglect and discrimination. In a transdiagnostic sample, paranoia and grandiosity were positively related to interpersonal trauma and unrelated to non-interpersonal trauma; these relationships were significantly mediated by impaired sleep.^23^ The association between disorganised behaviour and trauma was not clear in the literature.

The association between persecutory delusions, bullying and intimidation, discrimination and emotional neglect is in agreement with other studies.^24,25^

In our study, soliloquy was associated with witnessing violence at home. In some participants, soliloquy was identified in their medical file, and other patients reported it themselves. For the one who reported it, it is difficult to establish if it was soliloquy or hallucination of soliloquy.^26^ The association between soliloquy and domestic violence was not found in the literature.

Thematic associations were found between trauma and life events and positive psychotics symptoms such as hallucinations (olfactory, tactile, auditory verbal, somaesthetic) and delusions (death threat, suspiciousness, persecutory delusions, delusion of control, spiritual ideas). Thematic associations were also found between post-traumatic symptoms and positive psychotic symptoms. This is in agreement with other studies.^27,28^

In our study, delusion of life threat with altered behaviour to protect oneself was presented by two participants exposed to deliberate life threat. These participants reported both physical abuse, emotional abuse and emotional neglect. In a cross-sectional study, high perceived life threat was associated with post-traumatic stress disorder.^29^ We hypothesise that exposure to life threats induced deliberately might favour delusion of life threat.

### Time of exposure and cumulative exposure

In the study, some life and trauma events were preferentially associated with subtypes of positive psychotic symptoms.

The period of exposure to domestic violence in the population was between 5 and 13 years old, which corresponds to the suggested period of brain sensitivity to childhood trauma and adversity.^30^ In our sample, exposure to domestic violence was mainly exposure to verbal violence. We hypothesise that exposure to domestic verbal violence could have an impact on regions involved in the control of own-voice soliloquy, the inhibition of motor commands and first-person and third-person perspective.^31^

The period of exposure to childhood sexual abuse was between 10 and 12 years old. The pathway of genesis to a psychiatric disorder in response to childhood sexual abuse is complex and determine by multiple factors, such as genetic, epigenetic, neurobiological changes, neurochemical, synaptic changes and effects of neuroendocrine axes in response to stress.^32^ In our sample, exposure to sexual abuse before the age of 16 years was associated with memory perturbation, imposed visual mental imagery, anger, tactile and auditory-verbal hallucinations, persecutory and grandiose ideas, and delusion of control. Our finding is compatible with the hippocampal abnormalities found in neuroimaging studies,^33^ possibly switching off control over the limbic system, which then exacerbates emotional and post-traumatic stress disorder-related symptoms^34^ in response to sexual abuse. The symptoms of our participants who reported sexual abuse are congruent with the effect on the cerebellar vermis, a structure intricately linked to multisensory integration and limbic activation.^35^

### Participants’ narrative and causal theories

Most participants developed causal theories of positive psychotic symptoms including life and trauma events. Their theories including trauma and life events were compatible with theoretical models proposed in the literature concerning the association between trauma and psychosis: stress sensitivity,^36^ dissociation,^37^ self-disgust as a mediating factor,^38^ social pathways such as attachment disruption and social rank,^39^ and negative schemas about others and self.^40^

Most participants found a narrative interview about the link between trauma and life events and positive psychotic symptoms relevant and helpful. Such interviews allow individuals to be validated in their emotional experience, and to adopt an agent posture in which they develop an understanding of their experience in their personal lives and integrate their life experiences.

### Strengths and limitations

The study gains its strengths from the combination of in-depth qualitative analysis and exploratory cluster and matrix coding analyses. To our knowledge, this is the first study to apply this method to an FEP population.

All participants lived in Greater Lyon, which is mainly urban, and were involved in a FEP programme either in the in-hospital unit or in the out-patient programme with multidisciplinary care. Data may not reflect the views of people in other areas of France and outside FEP services. We recognise that several analyses are based on a small number of observations, which limits our ability to draw general conclusions. In particular, the data linking specific positive symptoms to certain types of trauma are based only on three participants. However, we felt it relevant to mention them, as they may inspire more systematic and quantitative explorations for this type of association.

Future research should consider testing the hypothesis derived from the exploratory cluster analysis and matrix coding analysis, and explore the neuroanatomy and the cognitive mechanisms underlying or mediating trauma and life events and positive psychotic symptoms.

In conclusion, our participants described a distressing experience of positive psychotic symptoms and multiple causal theories of their symptoms, often linking it with life and trauma events. Our findings point to the potential benefits of interviews exploring their symptoms considering their life and trauma events.

## Data Availability

The data that supports the findings of this study are available from the corresponding author, F.H., upon reasonable request.

## Appendix 1. Semi-structured interview guide

**Table:**

Would you be willing to talk about your first psychological difficulties?	
**Context**	**Question 1**: What was going on in your life when the difficulties that led you to be monitored and admitted to hospital arose? What was the context?
**Positive psychotic symptoms experience**	**Question 2**: What were the difficulties or symptoms that appeared at that time? Follow-ups: Could you elaborate on this? And clarify this? If it helps, try to think of a time when the symptoms were strongest or most present. At that time, what was occupying your mind most of the time? Were you preoccupied with something? Did you have many thoughts at the same time? Can you describe them for me? During this period of crisis, what was your state of mind? What were your emotions? How did you feel? What was your opinion of yourself at the time? Had you changed in any way? Had your behaviour changed? Did you do anything out of character or that you had not done before? Have those around you commented on your behaviour? If so, in what way? Could you elaborate? How did those close to you react to this change in your behaviour? Were your habits and activities different? At that time, what did you think of the world and of other people in general (e.g. your loved ones, your colleagues, people in the street)? Did you notice any changes in others, in the world or in your environment? Did you have any other difficulties (e.g. getting organised, communicating or doing things you used to do)? Can you elaborate? How was it more complicated? Did you have the impression that your thoughts and feelings were controlled by something outside you? (As if your thoughts didn’t belong to you or were controlled by an outside force). What about your actions? Did you feel dispossessed or no longer in control of yourself (thoughts, actions, feelings)? Did you notice any sensory changes (visual, auditory, smell, taste, touch or other sensations)? Can you describe them to me? In the case of voices: what were they saying? What did you hear? What emotions did they evoke? Were there things that increased or decreased your symptoms? What made the symptoms more or less intense (e.g. location, situation, what you were doing, thoughts, substances)?
**Link with past trauma or life events**	**Question 3:** Do your symptoms remind you of anything you’ve experienced before (before the first difficulties)? Follow-ups: Do your symptoms remind you of an event or something significant from your past? Or of things or people you’ve known? If so, can you tell me about them?
**Participants’ theories**	**Question 4:** Have you identified anything in your life that may have weakened you (these may have been minor at the time) from birth to before the first difficulties? Follow-ups: In your opinion, could this have contributed to your difficulties? If so, in what way?
**Relevance of the interview**	**Question 5:** Do you think that talking about the links between your personal history and your symptoms could help you? If so, how?
**Traumatic revival**	**Question 6** (if yes to TALE question 18): You may have told us about a difficult start to care (e.g. in the emergency room or during your hospital stay). Did this have an impact on your symptoms? Has it awakened any previous unpleasant memories or feelings?
